# Supplementary material for: Simarouba berteroana Krug & Urb. Extracts and Fractions Possess Anthelmintic Activity Against Eggs and Larvae of Multidrug-Resistant Haemonchus contortus
Source: Vet Sci. 2025 Jan 23;12(2):90. doi: 10.3390/vetsci12020090 (PMC11861957; doi:10.3390/vetsci12020090)
Supplement: Supplementary file 1 [file vetsci-12-00090-s001.zip › Suplementary_Material_Table_S3 20.12.24.pdf]

Table S3. Percentage (mean and standard deviation) of eggs and hatched larvae of *Haemonchus contortus* Kokstad exposed to extracts and fractions of *Simarouba berteroana* (1st & 2nd collections) exhibiting morphological alterations.

Extract/ Fraction	**Sb1**							**Sb2**						
	# Eggs	%ME	%LFE	# L_1	%L_1Alt-1	%L_1Alt-2	%L_1Alt-3	# Eggs	%ME	%LFE	# L_1	%L_1Alt-1	%L_1Alt-2	%L_1Alt-3
Crude extract (Sb1, mg/mL)														
3	101 (9,23)	30.50 (4.78)	69.5 (4.78)	0.17 (0.41)	0.0	-	-	101 (9.23)	30.5 (4.78)	69.5 (4.78)	0.167 (0.41)	0.0	-	-
2.5	102.5 (25.45)	26.48 (5.18)	73.52 (5.18)	0.94 (1.06)	0.0	0.0	0.0	117.29 (14.97)	15.15 (2.89)	84.85 (2.89)	3.29 (2.14)	0.0	0.0	0.0
2	77.93 (15.5)	30.64 (8.0)	69.36 (8.0)	37.33 (19.12)	4.89 (4.42)	0.6 (1.6)	0.80 (2.13)	56.85 (16.28)	32.71 (6.4)	67.29 (6.4)	91 (30.5)	9.03 (6.28)	0.33 (1.46)	0.0
1.5	40.65 (12.75)	40.15 (7.3)	59.85 (7.3)	84.41 (12.65)	21.58 (7.46)	0.58 (1.29)	0.18 (0.53)	35.06 (16.48)	44.25 (8.12)	55.75 (8.12)	122.28 (16.55)	34.25 (16.41)	0.46 (1.12)	0.0
1	26 (5.9)	51.28 (8.61)	48.72 (8.61)	79.27 (12.04)	38.03 (8.63)	-	-	27.21 (7.16)	44.03 (8.65)	55.97 (8.65)	131.93 (11.27)	38.62 (18.96)	0.0	0.0
0.5	18.89 (9.57)	91.74 (7.56)	8.26 (7.56)	105 (15.95)	14.96 (6.71)	0.0	0.54 (1.03)	13.21 (3.04)	89.07 (8.27)	10.93 (8.27)	147.14 (18.51)	12.5 (8.38)	0.0	0.28 (0.74)
0.25	15 (5.53)	95.76 (3.64)	4.24 (3.64)	118.09 (15.07)	0.52 (0.5)	0.0	0.31 (0.66)	9.86 (3.0)	87.95 (7.91)	12.05 (7.91)	152.86 (18.84)	0.69 (0.64)	0.0	0.0
HexFr (mg/mL)														
16	6.25 (4.03)	100.0	0.0	47.25 (10.63)	0.0	0.0	4.58 (9.17)	4 (1.83)	100.0	0.0	33.25 (14.55)	0.0	0.0	5.83 (11.67)
8	8.5 (3.11)	95.45 (9.09)	4.54 (9.09)	117.75	0.0	0.0	3.1 (6.19)	13.14 (3.09)	82.36 (19.21)	17.64 (3.03)	72.86 (11.03)	0.0	1.75 (2.93)	0.0
4	2.25 (0.83)	100.0	0.0	129.5 (7.37)	0.0	0.0	0.0	13.1 (9.0)	86.17 (14.65)	13.83 (14.65)	100.7 (19.41)	0.15 (0.48)	1.25 (2.18)	0.19 (0.4)
2	5.88 (4.67)	99.17 (2.36)	0.83 (2.36)	94.75 (26.14)	0.0	0.0	0.0	13.4 (7.59)	85.45 (7.45)	14.55 (7.45)	113.3 (31.19)	0.0	0.77 (1.71)	0.0
1	6.57 (4.35)	98.41 (4.2)	1.59 (4.2)	97.43 (21.44)	0.0	0.0	0.28 (0.48)	9.09 (6.35)	97.04 (6.11)	2.96 (6.11)	105.45 (39.84)	0.0	0.0	0.0
0.5	2.25 (0.43)	100.0	0.0	127 (10.93)	0.0	0.0	0.0	15.63 (6.0)	93.45 (6.85)	6.55 (6.85)	156.13 (30.06)	0.17 (0.32)	0.0	0.0
EtAcFr (mg/mL)														

3	185.86 (8.98)	15.9 (2.69)	84.1 (2.69)	185.86 (19.68)	0.0	0.0	0.0	107 (6.96)	23.54 (6.36)	76.46 (6.36)	0.0	0.0	0.0	0.0
2.5	120.71 (56.37)	18.19 (6.64)	81.81 (6.64)	34.86 (35.25)	0.89 (1.82)	0.21 (0.72)	0.83 (2.89)	-	-	-	-	-	-	-
2	134.42 (16.68)	12.66 (1.52)	87.34 (1.52)	63.33 (32.24)	2.34 (5.12)	1.06 (1.32)	0.0	170.63 (33.7)	10.25 (2.85)	89.75 (2.85)	7 (13.22)	0.0	0.0	4.99 (11.56)
1.5	72.57 (5.77)	17.13 (4.08)	82.87 (4.08)	132.71 (84.0)	13.12 (3.19)	5.8 (1.91)	0.87 (1.46)	65.91 (20.35)	30.51 (6.11)	69.49 (6.11)	131.91 (35.9)	8.51 (4.62)	7.72 (6.4)	1.5 (2.75)
1	56.09 (8.65)	28.9 (5.09)	71.1 (5.09)	146.18 (34.61)	34.02 (7.66)	6.61 (3.22)	0.79 (2.04)	57 (14.05)	27.64 (4.38)	72.36 (4.38)	144.83 (28.67)	15.4 (5.59)	12.69 (3.08)	1.74 (2.05)
0.5	18.33 (5.21)	78.72 (7.16)	21.28 (7.16)	197.67 (24.51)	22.1 (6.17)	1.02 (1.16)	0.15 (0.4)	20.33 (5.91)	64.01 (9.8)	35.99 (9.8)	195.08 (24.21)	22.3 (6.1)	6.75 (3.52)	0.82 (0.86)
0.25	12.45 (4.91)	95.48 (4.78)	4.52 (4.78)	215.91 (16.38)	4.81 (2.62)	0.15 (0.35)	0.16 (0.35)	14.08 (4.72)	94.79 (6.38)	5.21 (6.38)	204.25 (47.32)	4.96 (3.93)	1.0 (1.19)	2.59 (3.06)
isobFr (mg/mL)														
3	153.92 (30.78)	33.56 (4.34)	66.44 (4.34)	0.0	0.0	0.0	0.0	-	-	-	-	-	-	-
2.5	153.58 (35.6)	29.27 (3.98)	70.73 (3.98)	17.08 (16.86)	1.91 (3.07)	0.0	0.0	156.15 (51.46)	44.97 (7.42)	55.03 (7.42)	0.0	0.0	0.0	0.0
2	127.69 (34.19)	28.47 (4.89)	71.53 (4.89)	51.81 (24.18)	5.53 (4.02)	2.74 (4.02)	1.78 (2.31)	167.65 (38.21)	38.24 (4.58)	61.76 (4.58)	2.41 (5.39)	0.0	0.57 (1.6)	0.0
1.5	77.88 (17.8)	30.29 (5.28)	69.71 (5.28)	104.53 (32.05)	30.86 (11.62)	8.65 (4.14)	0.74 (2.09)	97.71 (21.37)	33.13 (5.29)	66.87 (5.29)	77.35 (27.71)	14.42 (8.73)	1.94 (2.21)	15.71 (19.35)
1	67 (10.73)	35.85 (6.49)	64.05 (6.49)	126 (12.25)	55.87 (6.91)	3.1 (2.21)	0.72 (1.97)	59.27 (9.03)	38.14 (4.93)	61.86 (4.93)	119.09 (12.19)	50.40 (18.06)	2.95 (2.3)	1.64 (3.47)
0.5	24.43 (6.62)	75.3 (5.26)	24.72 (5.26)	162.5 (28.38)	31.57 (13.46)	1.08 (0.86)	1.14 (2.03)	21.33 (2.16)	88.68 (5.41)	11.32 (5.41)	162.33 (21.49)	30.59 (15.22)	0.92 (0.46)	1.42 (2.05)
0.25	17.55 (4.34)	97.17 (4.62)	2.83 (4.62)	176.27 (17.32)	3.52 (1.97)	0.32 (0.55)	2.18 (278)	17.67 (4.03)	96.97 (7.42)	3.03 (7.42)	151.5 (30.79)	2.06 (1.61)	0.0	0.96 (1.02)
HalcFr mg/mL														
3	-	-	-	-	-	-	-	100.81 (21.61)	39.98 (9.54)	60.02 (9.54)	33.19 (33.8)	0.78 (1.44)	10.56 (17.48)	0.0
2.5	-	-	-	-	-	-	-	99.06 (36.35)	41.71 (6.97)	58.29 (6.97)	63.44 (47.51)	5.38 (12.58)	9.19 (13.43)	0.05 (0.21)

2	103 (7.95)	24.56 (1.9)	75.44 (1.9)	0.0	0.0	0.0	0.0	66.5 (31.48)	49.12 (8.14)	50.88 (8.14)	94.55 (36.49)	5.95 (3.25)	4.19 (3.88)	1.58 (4.29)
1.5	112.78 (17.62)	23.05 (5.56)	76.95 (5.56)	1.89 (3.02)	0.0	0.0	0.0	32.47 (16.64)	46.57 (8.01)	53.43 (80.1)	141.37 (19.37)	21.32 (11.51)	5.47 (6.01)	0.37 (0.93)
1	59.4 (27.7)	42.04 (8.39)	57.96 (8.39)	73.1 (20.77)	13.1 (9.6)	2.61 (3.89)	0.31 (0.66)	22.73 (10.69)	76.3 (6.17)	23.7 (6.17)	128.5 (24.5)	23.77 (12.11)	1.27 (2.67)	0.97 (1.65)
0.75	24 (3.32)	66.99 (9.43)	33.01 (9.43)	90.83 (9.3)	53.53 (3.51)	0.0	0.0	14 (6.89)	-	-	-	-	-	-
0.5	23.5 (3.29)	74.16 (4.62)	25.84 (4.62)	109.58 (14.36)	50.8 (6.18)	0.07 (0.24)	0.64 (1.5)		92.99 (6.93)	7.01 (6.93)	155.63 (15.58)	3.46 (3.54)	0.04 (0.16)	0.81 (3.24)
0.25	15.78 (7.14)	93.81 (5.61)	6.19 (5.61)	114.89 (29.55)	4.09 (4.06)	0.08 (0.23)	1.0 (1.71)	12.23 (4.57)	97.16 (5.88)	2.84 (5.88)	157.31 (8.2)	0.59 (0.96)	0.0	0.25 (0.73)
0.125	16.4 (3.78)	98.18 (4.07)	1.81 (4.07)	132.2 (16.71)	0.13 (0.29)	0.0	0.48 (1.08)	-	-	-	-	-	-	-
NC DMSO														
0.5%	2.75 (2.99)	95.83 (12.39)	4.17 (12.39)	151.07 (29.49)	0.0	0.0	0.39 (1.68)							
Thiabendazole														
0.1 mg/mL	99.54 (61.48)	99.82 (0.8)	0.18 (0.8)	4.26	0.0	0.0	0.0							

Eggs: total number of eggs counted; %ME: percentage of morulated eggs; %LFE: percentage of eggs with L_1 formed; #L1: total 1st-stage larvae of *H. contortus* counted; %L_1Alt-1: percentage hatched L_1 eviscerated or with disrupted cuticle and tissue release; %L_1Alt-2: percentage hatched L_1 with detachment of cuticle from the internal subjacent tissue; %L_1Alt-3: percentage of L1 with adhesions of extract material to the cuticle in the cuticle with external alterations. NC: negative control with distilled water and 0.5% Dimethylsulfoxide (DMSO).
