# Supplementary material for: Simarouba berteroana Krug & Urb. Extracts and Fractions Possess Anthelmintic Activity Against Eggs and Larvae of Multidrug-Resistant Haemonchus contortus
Source: Vet Sci. 2025 Jan 23;12(2):90. doi: 10.3390/vetsci12020090 (PMC11861957; doi:10.3390/vetsci12020090)
Supplement: Supplementary file 1 [file vetsci-12-00090-s001.zip › Suplementary_Material_Table_S1_and_S2_20.12.24.pdf]

Table S1. LC-MS analyses of the metabolites detected in the crude extracts and hydroalcoholic fractions of *Simarouba berteroana* (1st & 2nd collections).

Tentative compound annotation	Final ID[a]	RT[b]	M+H[c]	Neutral *m/z*[d]	MF[e]	Natural product class	Isolated from Genus / Family / Specie	Peak area[f] (x10^5) in each extract/fraction			
								Sb1	Sb1-halcFr	Sb2	Sb2-HalcFr
Glaucarubolone	4	9.7	395	394.1	$C_{20}H_{26}O_8$	Glycosylated quassinoids	Genus	0.00	0.00	0.497	3.915
Glaucarubol-15-Deoxy, 2-ketone	3	10.8	379	378.1	$C_{20}H_{26}O_7$	Quassinoids	Family	173.0	6.97	197.0	326.0
Simaroubin E	2	10.8	343	342.1	$C_{22}H_{30}O_3$	Terpenoid quinones	Genus	0.00	0.00	0.569	6.61
Javacarboline	1	12.9	321	320.0	$C_{20}H_{20}N_2O_2$	Carboline alkaloids	Family	3.5	0.00	0.00	0.00
Glaucarubinone; Glaucarubol-2-Ketone (2-hydroxy-2-methylbutanoyl)	18	14.1	495	494.2	$C_{25}H_{34}N_{10}$	Quassinoids	Family	3.31	17.2	2.9	16.8
Ailanquassin A-Dihydroxy	5	21.3	397	396.2	$C_{19}H_{24}O_9$	Quassinoids	Family	53.2	0.00	5.01	0.00
Amarolide-2-Glucopyranoside	23	21.3	527	526.4	$C_{26}H_{38}O_{11}$	Glycosylated quassinoids	Family	0.00	0.00	36.6	0.00
Ailanthus quassinoid 1	12	24.1	445	444.2	$C_{25}H_{32}O_7$	Quassinoids	Family	0.00	0.00	37.0	0.00
Glaucarubol; 2-Ketone-glucopyranoside	25	26.8	557	556.4	$C_{26}H_{36}O_{13}$	Glycosylated quassinoids	Genus	0.00	0.00	12.8	0.00
Glaucarubol 15-Glucopyranoside	29	28.1	559	558.4	$C_{26}H_{38}O_{13}$	Glycosylated quassinoids	Genus	2.1	0.00	4.23	0.00
24,25-Epoxy-6,23-dihydroxytirucall-8-en-3-one	15	28.6	473	472.3	$C_{30}H_{48}O_4$	Phytosterol lipids	Genus	3.42	0.00	0.00	0.00
Tirucalla-7,24-dien-3-one (stigmasta-7,22-dien-3)	9	32.1	425	424.2	$C_{30}H_{48}O$	Phytosterol lipids	Genus	39.7	82.6	61.9	19.7
Ailanquassin A-Dihydroxy	6	32.1	397	396.3	$C_{19}H_{24}O_9$	Quassinoids	Family	23.6	0.00	0.00	0.00
3-Hydroxytirucalla-7,24-dien-26-oic acid-3-Ketone	13	32.6	455	454.3	$C_{30}H_{46}O_3$	Phytosterol lipids	Genus	0.00	0.00	6.01	0.0
Glaucarubol; 15-(2-Acetoxy-2-methylbutanoyl)	24	32.7	539	538.4	$C_{27}H_{38}O_{11}$	Quassinoids	Genus	13.1	0.00	17.0	0.00
21,23,24,25-Diepoxytirucall-7-ene-3,21-diol-3-Ketone	14	33.4	471	470.3	$C_{30}H_{46}O_4$	Phytosterol lipids	Genus	13.2	0.00	4.13	0.00
3-Oxotirucalla-7,24-dien-21-al	11	33.7	439	438.3	$C_{30}H_{46}O_2$	Phytosterol lipids	Genus	0.00	0.00	10.1	0.00
Glaucarubol 15-Glucopyranoside	28	33.8	559	558.4	$C_{26}H_{38}O_{13}$	Glycosylated quassinoids	Genus	61.9	0.00	239.0	0.00

Brucein E; 2-Ketone, 15-benzoyl	21	34.1	515	514.4	$C_{27}H_{30}O_{10}$	Quinolone quassinoids (terpenoid quinones)	Family	0.00	0.00	32.3	0.00
Javanicin D	33	34.6	617	616.5	$C_{32}H_{40}O_{12}$	Naphthoquinones	Family	10.1	0.00	130.0	0.00
Simaroubin B	34	35.3	637	636.4	$C_{37}H_{48}O_{9}$	Terpenoid quinones	Genus	50.5	657.0	38.6	31.6
Glaucarubol; 2-Ketone-glucopyranoside	26	35.5	557	556.4	$C_{26}H_{36}O_{13}$	Glycosylated quassinoids	Genus	21.6	0.00	14.3	0.00
Glaucarubol-Benzoyloxy-15-deoxy-2-glucopyranoside	35	36.1	662	661.5	$C_{33}H_{42}O_{14}$	Glycosylated quassinoids	Family	16.9	0.00	0.00	0.00
5-Hydroxycanthin-6-one-Glucopyranosyl-glucopyranoside	30	36.8	561	560.5	$C_{26}H_{28}N_{2}O_{12}$	Glycosylated canthin-6-one alkaloids	Family	2.84	0.00	11.0	0.00
Brucein E; 2-Ketone, 15-benzoyl	22	37.2	515	514.4	$C_{27}H_{30}O_{10}$	Quinolone quassinoids (terpenoid quinones)	Family	59.4	0.00	15.8	0.00
Glaucarubol-13,18-Didehydro-2-ketone-(2-hydroxy-2-methylbutanoyl)	17	38.2	493	492.4	$C_{25}H_{32}O_{10}$	Quassinoids	Genus	105.0	0.00	19.4	0.00
Brucein K; Brucein E	7	40.5	413	412.3	$C_{20}H_{28}O_{9}$	Quinolone quassinoids (terpenoid quinones)	Family	26.5	3.32	0.00	0.00
Brucein K; Brucein E	8	41	413	412.6	$C_{20}H_{28}O_{9}$	Quinolone quassinoids (terpenoid quinones)	Family	2.31	11.7	59.8	2.25
Brucein E; 2-Ketone, 15-benzoyl	20	41.9	515	513.6	$C_{27}H_{30}O_{10}$	Quinolone quassinoids (terpenoid quinones)	Family	51.6	0.00	121.0	18.5
5,9-Dihydroxycanthin-6-one-glucopyranoside	10	41.9	429	428.2	$C_{21}H_{20}N_{2}O_{8}$	Glycosylated canthin-6-one alkaloids	Specie	15.9	0.00	2.83	0.00
Glaucarubol; 2-Ketone-glucopyranoside	27	42.3	557	556.5	$C_{26}H_{36}O_{13}$	Glycosylated quassinoids	Genus	2.65	7.64	36.6	3.35
Simaroubin A	16	42.8	482	480.6	$C_{30}H_{40}O_{5}$	Terpenoid quinones	Genus	5.64	0.00	0.00	0.00
Bruceanol G	32	44.3	608	607.5	$C_{30}H_{40}O_{13}$	Quassinoids	Family	6.3	0.00	0.00	0.00
Glaucarubinone; Glaucarubol-2-Ketone (2-hydroxy-2-methylbutanoyl)	19	44.8	496	494.8	$C_{25}H_{34}O_{10}$	Quassinoids	Genus	0.00	0.00	2.97	5.71
5-Hydroxycanthin-6-one-Glucopyranosyl-glucopyranoside	31	46.6	562	560.6	$C_{26}H_{28}N_{2}O_{12}$	Glycosylated canthin-6-one alkaloids	Family	0.00	0.000	14.8	0.00

[a]ID: annotations by dnp.chemnetbase.com (2024); [b]RT: retention Time; [c]M+H: mass of the ionized protonated molecule; [d]Molecular weight, [e]MF: molecular formula. [f]Peak intensity: peak area of *m/z* at its respective retention time. Sb1 and Sb1HalcFr: crude extract and the hydroalcoholic fraction of *S. berteroana* (1st collection), respectively, Sb2 and Sb2-HalcFr: crude extract and the hydroalcoholic fraction of *S. berteroana* (2nd collection), respectively.

Table S2. Molecular structure and biological sources of metabolites detected in the LC-MS analyses of the crude extracts and hydroalcoholic fractions of *Simarouba berteroana* (1st & 2nd collections).

Tentative compound annotation	Final ID[a]	Natural product class	SMILES	MF[b]	Molecular structure	Isolated from Genus / Family / Specie	Biological Source
Glaucarubolone	4	Glycosylated quassinoids	CC1C2C(C(=O)OC3C24COC(C1O)(C4C5(C(C3)C(=CC(=O)C5O)C)C)O)O	$C_{20}H_{26}O_8$	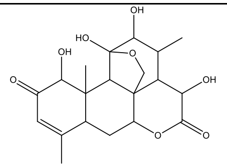	Genus	*Quassia simarouba* (Simaroubaceae)
Glaucarubol-15-Deoxy, 2-ketone	3	Quassinoids	CC1C(O)C2(O)OCC34C2C2(C)C(O)C(=O)C=C(C)C2CC3OC(=O)CC14	$C_{20}H_{26}O_7$	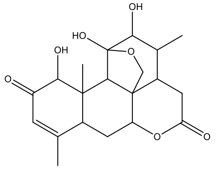	Family	Isol. from <i>*Hannoa klaineana*</i>, <i>*Ailanthus integrifolia*</i>, <i>*Simaba multiflora*</i>, <i>*Soulamea tomentosa*</i>, <i>*Castela tortuosa*</i>, <i>*Simaba cedron*</i>, <i>*Ailanthus altissima*</i> and others (Simaroubaceae)
Simaroubin E	2	Terpenoid quinones	CC12C=CC(=O)OC(C)(C)C1CC(=O)C1(C)C2CCC23CC12CCC3	$C_{22}H_{30}O_3$	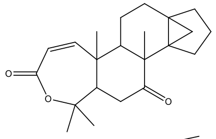	Genus	Constit. of <i>*Simarouba amara*</i> (Simaroubaceae)
Javacarboline	1	Carboline alkaloids	CCc1c(C)c[n+]2C(Cc3c([nH]c4ccccc34)-c2c1C)C([O-])=O	$C_{20}H_{20}N_2O_2$	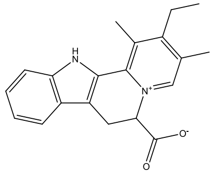	Family	Alkaloid from the stem of <i>*Picrasma javanica*</i> (Simaroubaceae)
Glaucarubinone; Glaucarubol-2-Ketone (2-hydroxy-2-methylbutanoyl)	18	Quassinoids	CCC(C)(O)C(=O)OC1C2C(C)C(O)C3(O)OCC22C3C3(C)C(O)C(=O)C=C(C)C3CC2OC1=O	$C_{25}H_{34}N_{10}$	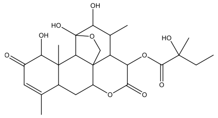	Family	*Simarouba glauca* (Simaroubaceae)

Ailanquassin A-Dihydroxy	5	Quassinoids	CC1C(O)C2(O)OCC34C(CC(C)(C5OC(=O)C=C5C)C23)OC(=O)C(O)C14O	$C_{19}H_{24}O_9$	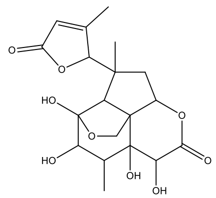	Family	Constit. of <i>*Eurycoma longifolia*</i>
Amarolide-2-Glucopyranoside	23	Glycosylated quassinoids	CC1CC(OC2OC(CO)C(O)C(O)C2O)C(=O)C2(C)C1CC1OC(=O)CC3C(C)C(=O)C(O)C2C13C	$C_{26}H_{38}O_{11}$	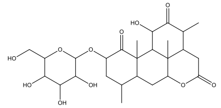	Family	Constit. of <i>*Ailanthus altissima*</i>(Simaroubaceae)
Ailanthus quassinoid 1	12	Quassinoids	CCC(C)C(=O)OC1C2C(C)C(O)C3(O)OCC22C3c3c(C)ccc(C)c3CC2OC1=O	$C_{25}H_{32}O_7$	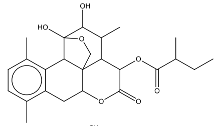	Family	Constit. of <i>*Ailanthus excelsa*</i> (Simaroubaceae)
Glaucarubol; 2-Ketone-glucopyranoside	25	Glycosylated quassinoids	CC1C(O)C2(O)OCC34C2C2(C)C(O)C(=O)C=C(C)C2CC3OC(=O)C(OC2OC(CO)C(O)C(O)C2O)C14	$C_{26}H_{36}O_{13}$	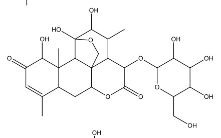	Genus	Constit. of <i>*Simarouba glauca*</i> and <i>*Castela marcophylla*</i> (Simaroubaceae)
Glaucarubol 15-Glucopyranoside	29	Glycosylated quassinoids	CC1C(O)C2(O)OCC34C2C2(C)C(O)C(O)C=C(C)C2CC3OC(=O)C(OC2OC(CO)C(O)C(O)C2O)C14	$C_{26}H_{38}O_{13}$	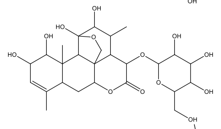	Genus	Constit. of <i>*Simarouba glauca*</i> (Simaroubaceae)
24,25-Epoxy-6,23-dihydroxytirucall-8-en-3-one	15	Phytosterol lipids	CC(CC(O)C1OC1(C)C)C1CCC2(C)C3=C(CCC12C)C1(C)CCC(=O)C(C)(C)C1C(O)C3	$C_{30}H_{48}O_4$	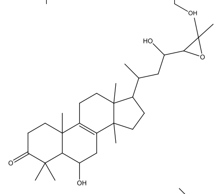	Genus	Constit. of <i>*Simarouba amara*</i> (Simaroubaceae)
Tirucalla-7,24-dien-3-one (stigmasta-7,22-dien-3)	9	Phytosterol lipids	CC(CCC=C(C)C)C1CCC2(C1(CCC3C2=CCC4C3(CCC(=O)C4(C)C)C)C)C	$C_{30}H_{48}O$	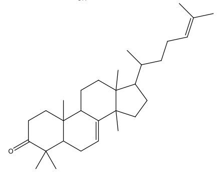	Genus	Constit. of <i>*Simarouba amara*</i> and <i>*Vismia laurentii*</i> (Simaroubaceae)

Ailanquassin A-Dihydroxy	6	Quassinoids	CC1C(O)C2(O)OCC34C(CC(C)(C5OC(=O)C=C5C)C23)OC(=O)C(O)C14O	$C_{19}H_{24}O_9$	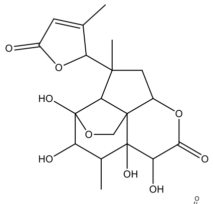	Family	Constit. of <i>*Eurycoma longifolia*</i>
3-Hydroxytirucalla-7,24-dien-26-oic acid-3-Ketone	13	Phytosterol lipids	CC(CC\C=C(/C)C(O)=O)C1CCC2(C)C3=CCC4C(C)(C)C(=O)CCC4(C)C3CCC12C	$C_{30}H_{46}O_3$	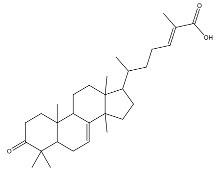	Genus	Constit. of <i>*Simarouba amara*</i> and <i>*Dysoxylum pettigrewianum*</i> (Simaroubaceae)
Glaucarubol; 15-(2-Acetoxy-2-methylbutanoyl)	24	Quassinoids	CCC(C)(OC(C)=O)C(=O)OC1C2C(C)C(O)C3(O)OCC22C3C3(C)C(O)C(O)C=C(C)C3CC2OC1=O	$C_{27}H_{38}O_{11}$	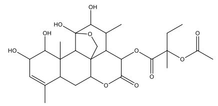	Genus	Constit. of <i>*Simarouba amara*</i>, <i>*Pierreodendron kerstingii*</i>, <i>*Odyendea gabonensis*</i> and <i>*Perriera*</i> sp. (Simaroubaceae)
21,23,24,25-Diepoxytirucall-7-ene-3,21-diol-3-Ketone	14	Phytosterol lipids	CC1(C)OC1C1CC(C(O)O1)C1CCC2(C)C3=CCC4C(C)(C)C(=O)CCC4(C)C3CCC12C	$C_{30}H_{46}O_4$	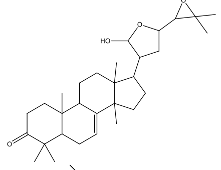	Genus	Constit. of <i>*Melia azedarach*</i>, <i>*Simarouba amara*</i>, <i>*Neochamaelea pulverata*</i> and <i>*Eurycoma longifolia*</i> (Simaroubaceae)
3-Oxotirucalla-7,24-dien-21-al	11	Phytosterol lipids	C/C(C)=C\CCC(C([H])=O)C1CCC2(C)C1(C)CCC3C2=CCC4C3(C)CCC(C4(C)C)=O	$C_{30}H_{46}O_2$	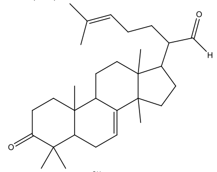	Genus	Constit. of <i>Simarouba amara</i> and <i>Owenia cepiodora</i> (Simaroubaceae)
Glaucarubol 15-Glucopyranoside	28	Glycosylated quassinoids	CC1C(O)C2(O)OCC34C2C2(C)C(O)C(O)C=C(C)C2CC3OC(=O)C(OC2OC(CO)C(O)C(O)C2O)C14	$C_{26}H_{38}O_{13}$	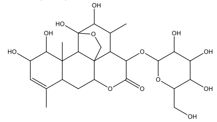	Genus	Constit. of <i>*Simarouba glauca*</i> (Simaroubaceae)
Brucein E; 2-Ketone, 15-benzoyl	21	Quinolone quassinoids (terpenoid quinones)	CC1=CC(=O)C(O)C2(C)C1CC1OC(=O)C(OC(=O)c3ccccc3)C3(O)C4(C)OCC13C2C(O)C4O	$C_{27}H_{30}O_{10}$	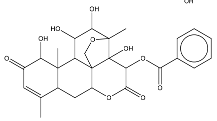	Family	Constit. of <i>Soulamea amara</i>

Javanicin D	33	Naphthoquinones	COC1CC2C(C)(O)C(OC(C)=O)C(OC(=O)c3ccc4OCOc4c3)C3C2(C)C(CC2CCC(OC(C)=O)C(=O)C32C)O1	$C_{32}H_{40}O_{12}$	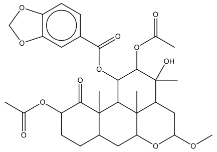	Family	Constit. of <i>*Picrasma javanica*</i> (Simaroubaceae)
Simaroubin B	34	Terpenoid quinones	C\C=C(/C)C(=O)OC1CC23CC2(CCC3C2=CC(OC2=O)C2OC2(C)C)C2(C)C(CC3C(C)(C=CC(=O)OC3(C)C)C12)OC(C)=O	$C_{37}H_{48}O_9$	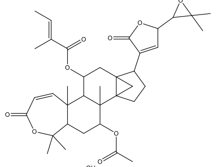	Genus	Constit. of <i>*Simarouba amara*</i> (Simaroubaceae)
Glaucarubol; 2-Ketone-glucopyranoside	26	Glycosylated quassinoids	CC1C(O)C2(O)OCC34C2C2(C)C(O)C(=O)C=C(C)C2CC3OC(=O)C(OC2OC(CO)C(O)C(O)C2O)C14	$C_{26}H_{36}O_{13}$	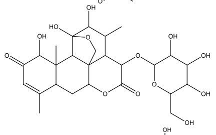	Genus	Constit. of <i>*Simarouba glauca*</i> and <i>*Castela marcophylla*</i> (Simaroubaceae)
Glaucarubol-Benzoyloxy-15-deoxy-2-glucopyranoside	35	Glycosylated quassinoids	CC1C(O)C2(O)OCC34C2C2(C)C(O)C(OC5OC(CO)C(O)C(O)C5O)C=C(C)C2C(OC(=O)c2ccccc2)C3OC(=O)CC14	$C_{33}H_{42}O_{14}$	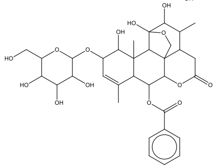	Family	Constit. of <i>*Ailanthus altissima*</i>(Simaroubaceae)
5-Hydroxycanthin-6-one-Glucopyranosyl-glucopyranoside	30	Glycosylated canthin-6-one alkaloids	OCC1OC(OCC2OC(Oc3cc4nccc5c6ccccc6n(c45)c3=O)C(O)C(O)C2O)C(O)C(O)C1O	$C_{26}H_{28}N_2O_{12}$	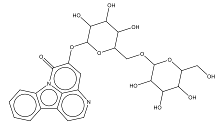	Family	Alkaloid from stems of <i>*Brucea javanica*</i> and root wood of <i>*Brucea mollis*</i> var. <i>*tonkinensis*</i> (Simaroubaceae)
Brucein E; 2-Ketone, 15-benzoyl	22	Quinolone quassinoids (terpenoid quinones)	CC1=CC(=O)C(O)C2(C)C1CC1OC(=O)C(OC(=O)c3ccccc3)C3(O)C4(C)OCC13C2C(O)C4O	$C_{27}H_{30}O_{10}$	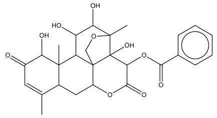	Family	Constit. of <i>*Soulamea amara*</i>
Glaucarubol-13,18-Didehydro-2-ketone-(2-hydroxy-2-methylbutanoyl)	17	Quassinoids	CCC(C)(O)C(=O)OC1C2C(=C)C(O)C3(O)OCC22C3C3(C)C(O)C(=O)C=C(C)C3CC2OC1=O	$C_{25}H_{32}O_{10}$	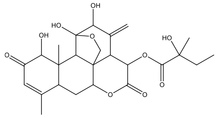	Genus	Constit. of <i>*Simarouba amara*</i> (Simaroubaceae)
Brucein K; Brucein E	7	Quinolone quassinoids (terpenoid quinones)	CC1=CC(O)C(O)C2(C)C1CC1OC(=O)C(O)C3(O)C(C)(O)C4OCC13C2C4O	$C_{20}H_{28}O_9$	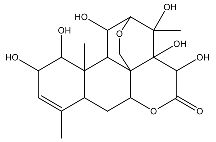	Family	Constit. of <i>*Brucea javanica*</i> (Simaroubaceae)

Brucein K; Brucein E	8	Quinolone quassinoids (terpenoid quinones)	CC1=CC(O)C(O)C2(C)C1CC1OC(=O)C(O)C3(O)C(C)(O)C4OCC13C2C4O	$C_{20}H_{28}O_9$	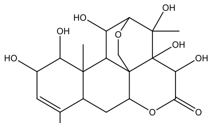	Family	Constit. of <i>*Brucea javanica*</i> (Simaroubaceae)
Brucein E; 2-Ketone, 15-benzoyl	20	Quinolone quassinoids (terpenoid quinones)	CC1=CC(=O)C(O)C2(C)C1CC1OC(=O)C(OC(=O)c3ccccc3)C3(O)C4(C)OCC13C2C(O)C4O	$C_{27}H_{30}O_{10}$	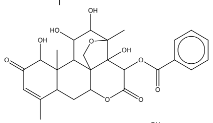	Family	Constit. of <i>*Soulamea amara*</i>
5,9-Dihydroxycanthin-6-one-glucopyranoside	10	Glycosylated canthin-6-one alkaloids	COc1ccc2c3ccnc4cc(OC5OC(CO)C(O)C(O)C5O)c(=O)n(c2c1)c34	$C_{21}H_{20}N_2O_8$	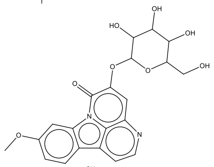	Specie	Alkaloid from the stem bark of <i>*Simarouba berteroana*</i> (Simaroubaceae)
Glaucarubol; 2-Ketone-glucopyranoside	27	Glycosylated quassinoids	CC1C(O)C2(O)OCC34C2C2(C)C(O)C(=O)C=C(C)C2CC3OC(=O)C(OC2OC(CO)C(O)C(O)C2O)C14	$C_{26}H_{36}O_{13}$	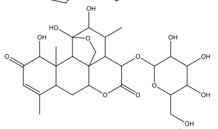	Genus	Constit. of <i>*Simarouba glauca*</i> and <i>*Castela marcophylla*</i> (Simaroubaceae)
Simaroubin A	16	Terpenoid quinones	CC1(C)OC1C1CC(=CO1)C1CCC23CC12CCC1C2(C)C=CC(=O)OC(C)(C)C2CC(=O)C31C	$C_{30}H_{40}O_5$	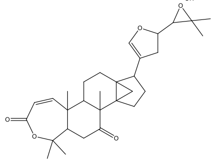	Genus	Constit. of <i>*Simarouba amara*</i>(Simaroubaceae)
Bruceanol G	32	Quassinoids	COC(=O)C12OCC34C1C(OC(=O)\C=C(/C)C(C)(C)OC(C)=O)C(=O)OC3CC1C(C)CC(=O)C(O)C1(C)C4C(O)C2O	$C_{30}H_{40}O_{13}$	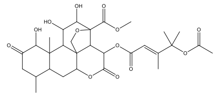	Family	Constit. of <i>*Brucea antidysenterica*</i> (Simaroubaceae)
Glaucarubinone; Glaucarubol-2-Ketone (2-hydroxy-2-methylbutanoyl)	19	Quassinoids	CCC(C)(O)C(=O)OC1C2C(C)C(O)C3(O)OCC22C3C3(C)C(O)C(=O)C=C(C)C3CC2OC1=O	$C_{25}H_{34}O_{10}$	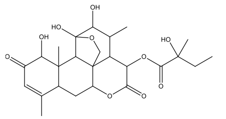	Genus	*Simarouba glauca* (Simaroubaceae)
5-Hydroxycanthin-6-one-Glucopyranosyl-glucopyranoside	31	Glycosylated canthin-6-one alkaloids	OCC1OC(OCC2OC(Oc3cc4nccc5c6ccccc6n(c45)c3=O)C(O)C(O)C2O)C(O)C(O)C1O	$C_{26}H_{28}N_2O_{12}$	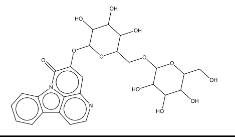	Family	Alkaloid from stems of <i>*Brucea javanica*</i> and root wood of <i>*Brucea mollis*</i> var. <i>*tonkinensis*</i> (Simaroubaceae)

[a]ID: annotations by dnp.chemnetbase.com (2024); [b]Molecular weight, [c]MF: molecular formula.
