# Supplementary figures and images for: Simarouba berteroana Krug & Urb. Extracts and Fractions Possess Anthelmintic Activity Against Eggs and Larvae of Multidrug-Resistant Haemonchus contortus
Source: Vet Sci. 2025 Jan 23;12(2):90. doi: 10.3390/vetsci12020090 (PMC11861957; doi:10.3390/vetsci12020090)

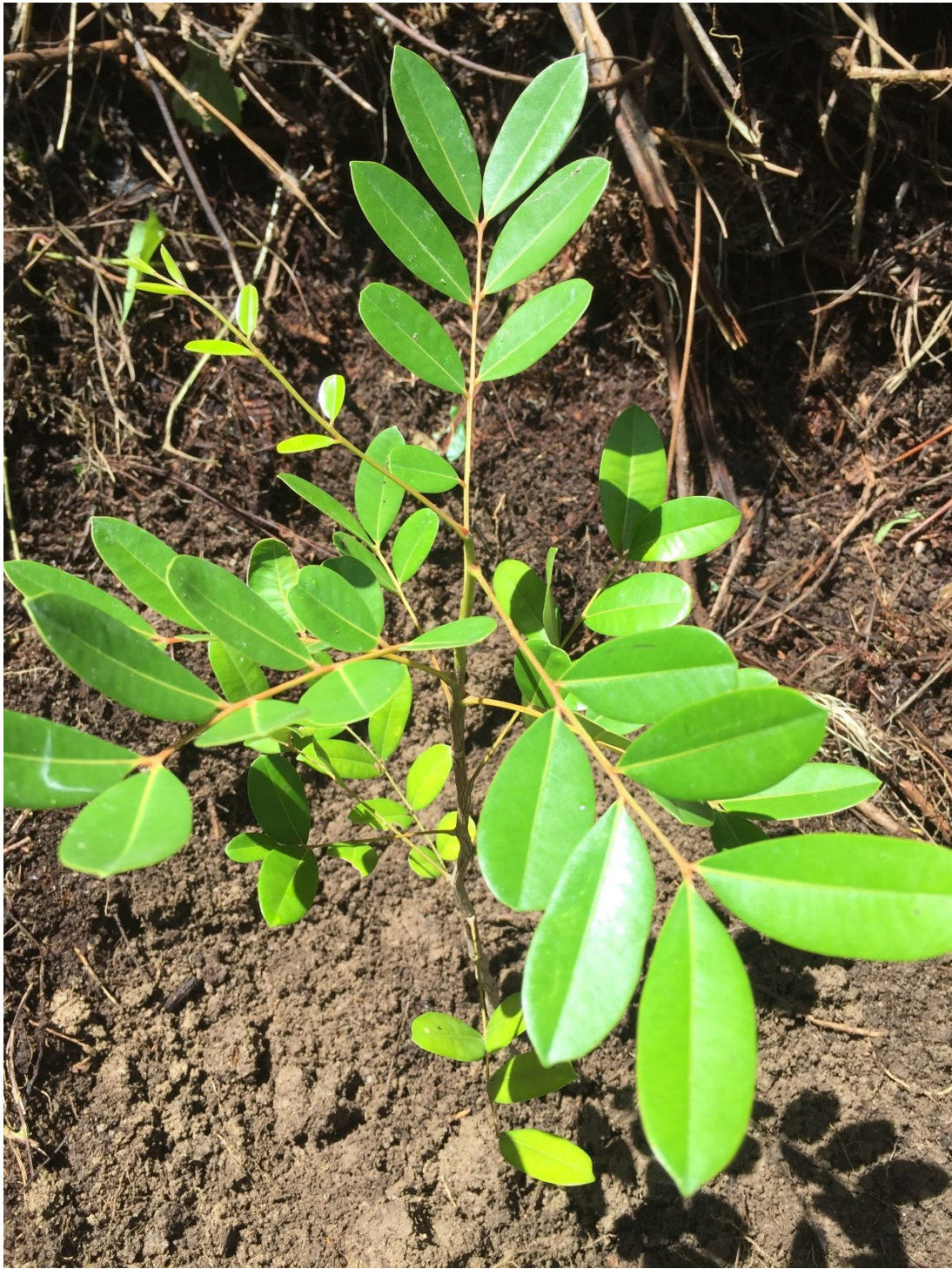

**Figure S1.** Young *Simarouba berteriana* plant cultivated in La Vega province, Dominican Republic.

Supplement: Supplementary file 1 [file vetsci-12-00090-s001.zip › Suplementary Figure SF1 S berteroana article 7.11.24.pdf]

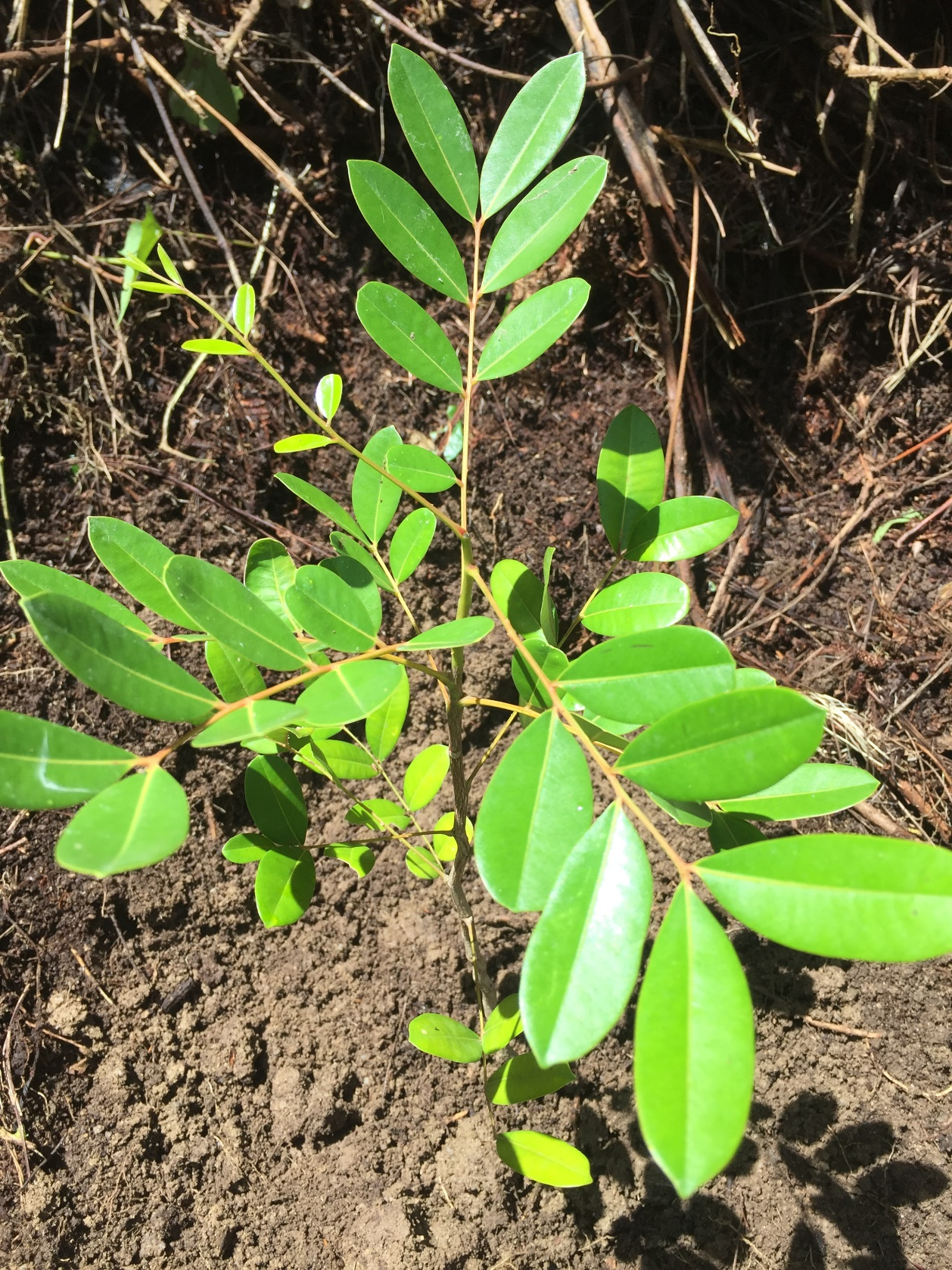

Supplement: Supplementary file 1 [file vetsci-12-00090-s001.zip › Young S berteroana article.tif]
